# The role of miRNAs as biomarkers in heterotopic ossification

**DOI:** 10.1530/EOR-22-0100

**Published:** 2024-12-02

**Authors:** Chen Xie, Xiao Liu, Wenbao Li, Zhaozhe Yao, Hongyue Men, Zongyu Li

**Affiliations:** 1 Trauma center, The 960th Hospital of PLA, Jinan, Shandong, China; 2 Department of Basic Medical Sciences, The 960th Hospital of PLA, Jinan, Shandong, China

**Keywords:** Heterotopic ossification, miRNA, pathogenesis, early diagnosis, treatment

## Abstract

- Fibrodysplasia ossificans progressiva and progressive osseous heteroplasia are genetic forms of heterotopic ossification (HO). Fibrodysplasia ossificans progressiva is caused by *ACVR1* gene mutations, while progressive osseous heteroplasia is caused by *GNAS* gene mutations.
- Nongenetic HO typically occurs after trauma or surgery, with an occurrence rate of 20–60%. It can also be observed in conditions such as diffuse idiopathic skeletal hyperostosis, spinal ligament ossification, ankylosing spondylitis, and skeletal fluorosis. The exact cause of nongenetic HO is not entirely clear.
- More than 100 types of miRNAs have been identified as being linked to the development of HO. Some miRNAs are promising potential biomarkers for traumatic HO and ossification of the posterior longitudinal ligament. These findings further emphasize the significant role miRNAs play in the pathogenesis and progression of bone disorders.
- Repeated investigations into the function of a specific miRNA are infrequent and yield inconsistent results, possibly because of variable experimental conditions.
- It is hypothesized that miRNAs can enhance osteogenesis for the management of fractures and bone defects. However, further research is required to validate this hypothesis.

## Introduction

Heterotopic ossification (HO) is characterized by the development of mature lamellar bone in soft tissues beyond the skeletal structure, encompassing muscles, tendons, and other soft tissues. This process is typically categorized into genetic and nongenetic forms (1). Genetic forms, exemplified by fibrodysplasia ossificans progressiva (FOP) and progressive osseous heteroplasia (POH), are associated with identifiable gene abnormalities, leading to a relatively clear understanding of their etiology and pathogenesis. In contrast, the pathogenesis of nongenetic HO remains incompletely elucidated (2).

Research has revealed that 80% of human posttranscriptional processes are controlled by miRNAs (3). Mature miRNAs interact with specific regions in the 3′ untranslated region (3′ UTR) of target mRNAs, leading to the inhibition of translation or degradation, which results in the downregulation of target mRNA expression and the execution of diverse and intricate regulatory roles (4). Additionally, a single miRNA can regulate numerous mRNAs, while a single mRNA can be targeted by multiple miRNAs, forming a sophisticated network of interactions (4).

Recently, several miRNAs have been recognized for their involvement in the control of gene expression related to osteogenic differentiation and the osteogenic process (5, 6). This study examines the categorization, causes, and mechanisms of HO, along with the influence of miRNA on HO, to propose novel strategies for the prevention and management of HO and other skeletal disorders.

## Genetic HO

### Fibrodysplasia ossificans progressiva

FOP is a rare genetic disorder with an incidence ranging from 0.036 per million in the Asia–Pacific region to 0.65 per million in North America (7). Patients with FOP present with congenital thumb or big toe deformity, followed by calcification in the shoulder joint during adolescence, resulting in restricted upper limb mobility. During adulthood, hip joint involvement leads to immobility and prolonged bed rest. Progressive spinal deformity, exacerbated by ossification of the thoracic cage, impairs respiratory function. Prolonged bed rest further increases susceptibility to pulmonary and urinary tract infections, ultimately endangering the patient's life (8, 9).

Studies have found that heterozygous missense mutations of activin A receptor type 1 (*ACVR1*) genes, which are located at 2q23–24, are the main cause of FOP (7). *ACVR1* is also the receptor of the bone morphogenetic protein type I (*BMP1*) (10). About 86–97% of FOP patients have an *ACVR1* point mutation (c.617G>A; p.R206H) (11, 12), and these patients are categorized as having classic FOP (13). A small number of patients have been found to have other site abnormalities, including c.982G>C/T/A, c.1067G>A, c.1124G>C, and c.590_592delCTT, and these patients are categorized as having FOP variants (13). All the aforementioned mutations occur in either the cytoplasmic juxtamembrane region, rich in glycine and serine residues, or the protein kinase domains, regions of the *ACVR1* receptor that are important for downstream signal transduction (13).

Surgical intervention often results in the progression of HO in FOP; therefore, it is generally not recommended for FOP patients (14). However, there have been reported cases where surgery has exhibited positive effects in alleviating the condition (15, 16, 17, 18). Palovarotene is an oral retinoic acid receptor γ agonist that acts by downregulating the bone morphogenetic protein (*BMP*) signaling pathway and activating the retinoic acid signaling pathway, thereby reducing the formation of new heterotopic bone in FOP. In 2023, the U.S. Food and Drug Administration approved palovarotene as a treatment for FOP, making it the only currently available approved treatment for FOP (19). Clinical studies have also reported that garetosmab (activin A inhibitor) can inhibit the occurrence and growth of HO in patients with FOP (20). However, rapamycin (an immunosuppressant) is ineffective in controlling the progression of FOP (21). Animal experiments have demonstrated that saracatinib (an *ACVR1* kinase inhibitor) can significantly inhibit the progression of HO in a mouse model of FOP (22).

In 2012, Mura *et al*. (23) used four software programs (TargetScan 5.1, PicTar, miRBase, and miRanda) to predict miRNAs that target the *ACVR1* 3' UTR sequence. They then validated these predictions by experimenting with three cell lines (HeLa, U2OS, and C2C12). Their findings indicated that miR-26a increases *ACVR1* mRNA expression, while miR-148b and miR-365 decrease *ACVR1* mRNA expression. In a separate investigation (24), three software programs (TargetScan 5.1, miRanda, and miRDB) identified 33 miRNAs that target *ACVR1*, including miR-148a. Experiments on HeLa cells confirmed that miR-148a decreases the mRNA and protein levels of *ACVR1*. Additionally, the miR-148a intervention resulted in a reduction in the mRNA expression of Id3, a crucial component of the *BMP* signaling pathway, providing further evidence of miR-148a's inhibitory impact on *BMP* signaling transduction. Furthermore, several studies have been conducted on the intervention of FOP mice using adeno-associated virus serotype 9 carrying artificial miRNA that targets *ACVR1*. The results demonstrated that the intervention was successful in both preventing and treating trauma-induced HO in FOP mice. This was achieved by specifically targeting activin A and its receptor *ACVR1* (25, 26). Despite the encouraging results of these studies, the application of such techniques in clinical trials has been delayed due to safety concerns (12).

### Progressive osseous heteroplasia

POH is a rare condition, with approximately 60 confirmed cases reported globally (27). HO in POH usually begins in the dermis or subcutaneous adipose tissue during infancy or early childhood, advancing to deeper connective tissues, muscles, tendons, and ligaments over time (27). This progressive ossification leads to significant stiffness and restriction in limb growth, contributing to the characteristic short stature commonly observed in individuals with POH (28). Interestingly, the majority of patients diagnosed with POH (in one study, 10 out of 12 cases) demonstrate a unilateral tendency for HO occurrence, with six cases on the left side and four cases on the right side (29).

The *GNAS* gene, which is located at 20q13.3, produces at least five transcripts, including *Gsα* and *XLαs* (30). Approximately 60% of POH cases are caused by paternally inherited *GNAS* exon heterozygous mutations, while approximately 17% of cases are caused by deletions in the c565–568 region. The majority of these mutations occur within exonic regions that are shared by *Gsα* and *XLαs* (31, 32, 33). In a small percentage of POH cases, abnormalities in the *GNAS* gene are not present, which leads to an unclear etiology (34).

*Gsα* and *XLαs* play important roles in skeletal development through the regulation of various signaling pathways, including the *cAMP-PKA*, *Wnt/β-catenin*, and Hedgehog pathways (35, 36). Mutations in *GNAS* can lead to inactivation of *Gsα* or loss of *XLαs* expression, resulting in disrupted bone formation and the development of osteochondrodysplasia (32, 33, 37). Gsα negatively regulates skeletal development through the *cAMP-PKA*, *Wnt/β-catenin*, and Hedgehog signaling pathways (35). *XLαs*, upon activation by *GPCR*, promoted *cAMP* production and shares similar functions with *Gsα* (36).

Effective treatment options for POH remain limited. Surgical excision of well-defined HO areas may provide relief from symptoms, but there is a risk of recurrence or complications (38). In severe cases involving functional contractures, amputation may be necessary (39, 40). Research has revealed that bisphosphonates, glucocorticoids, sodium thiosulfate (41), and everolimus (42) do not effectively alleviate POH symptoms. However, some studies have suggested that bisphosphonates may reduce bone turnover markers in POH patients, potentially delaying the onset of new HO (43).

Gómez-Carballa *et al*. (44) identified divergent clinical outcomes in twin sisters with a *GNAS* (565–568 del GACT) inactivation heterozygous mutation. One sister presented with invasive and disabling ectopic ossification, while the other sister exhibited minimal symptoms. Analysis of the blood transcriptome revealed significant variations in the expression levels of five miRNAs (miR-208b-5p, miR-556-5p, miR-518f-3p, miR-493-3p, and miR-106b-5p) between the siblings. Previous research has identified some indicators that were linked to osteogenic differentiation and bone tumor development. For instance, miR-208 has been demonstrated to impede *BMP2*-induced osteoblast differentiation by suppressing the expression of the target gene *Ets1* (45). Additionally, miR-556-5p, which is regulated by circ:0096041, has been linked to enhancing osteosarcoma proliferation (46), while miR-493 has been found to inhibit osteosarcoma proliferation and invasion by negatively modulating *SP1* (47).

## Diseases with HO

### Skeletal fluorosis

Skeletal fluorosis (SL) is a chronic metabolic bone disorder resulting from the excessive ingestion of fluoride. Prolonged consumption of water containing fluoride levels greater than 1.5 mg/L is the primary etiological factor of SL (48). SL is prevalent in more than 20 countries across Asia, Africa, and Latin America, including India, China, Thailand, Kenya, Egypt, Libya, Jordan, Mexico, and Argentina, making it a significant global public health concern (49). However, it is less common in Europe and North America (49).

The impact of fluoride on bone is bidirectional, with potential outcomes including bone sclerosis through increased bone mass or enhanced osteogenic activity, as well as bone resorption and the subsequent development of osteoporosis (50). The symptoms of fluorosis are determined based on the varying levels of fluoride consumed. Initially, individuals may experience minor joint or back pain, which can progress to osteosclerosis, osteochondrosis, and degenerative changes in joint cartilage with higher intake levels; further increases in dosage can result in osteoporosis (51). Studies dating back to the 1960s have explored the use of fluoride for treating osteoporosis, revealing that although fluoride can enhance axial bone density, it does not reduce the risk of spinal fractures or gastrointestinal issues (52, 53).

The diagnostic criteria for SL consist of several key components (51, 54): (i) residing in an endemic area for more than 2 years or displaying dental fluorosis, (ii) presenting with symptoms of bone and joint pain accompanied by functional limitations, and (iii) radiographic assessments demonstrating increased bone density in the pelvis and lumbar vertebrae. In severe cases, interosseous bridging and visible bone loss may be observed, suggestive of osteoporosis or osteomalacia (iv). The final criterion is urinary fluoride concentrations greater than the established normal thresholds.

The symptoms of SL exhibit similarities to those of rheumatoid arthritis, emphasizing the importance of accurate differentiation (51). Treatment approaches for SL predominantly focus on discontinuing exposure to high-fluoride settings, promoting fluoride excretion (through the use of vitamins C, D, and E), managing pain, and providing additional nutritional support. Surgical procedures may be necessary to address joint deformities (55).

Three research teams conducted comprehensive research on the role of miRNAs in the occurrence and development of SL. Deng *et al*. (56), using mouse osteoblasts treated with sodium fluoride as the research subject, discovered that miR-302d-3p, miR-6406, and miR-294-3p were closely associated with apoptosis via next generation sequencing (NGS also known as high-throughput sequencing) and quantitative PCR (qPCR). The second team found that miR-200c-3p negatively regulates osteogenesis by inhibiting the expression of the target gene noggin, based on the human osteosarcoma cell line SaoS2 treated with sodium fluoride (57). They also discovered that miR-21-5p positively regulates osteogenesis by inhibiting its target genes *PTEN* and *DKK2* (58). The third team initially used plasma from SL patients and healthy individuals as research subjects and identified 127 differentially expressed miRNAs via NGS (59). Subsequently, they further confirmed the role of these differential miRNAs in SL and discovered that fluoride could upregulate the expression of the target genes of these miRNAs by inhibiting the expression of miR-4755-5p (target gene *Cyclin D1*) (60), miR-122-5p (*CDK4*) (61), mir-7c-5p (*Cyclin D1* and *Wnt9a*) (62), and mir-486-3p (*Cyclin D1* and *TGF-β1*) (63), thereby promoting the proliferation and differentiation of human osteoblasts.

### Diffuse idiopathic skeletal hyperostosis

Diffuse idiopathic skeletal hyperostosis (DISH), also referred to as Forestier's disease, is characterized by the ossification of systemic ligaments and tendons, as well as the development of bone spurs (64). This condition primarily affects the spine, with the thoracic spine being the most commonly affected region, followed by the cervical and lumbar spines (65). DISH exhibits a higher prevalence in men, and its occurrence tends to increase with advancing age. Specifically, the incidence of DISH in men aged 50 and older is 25%, compared to 15% in women (66). Furthermore, in men aged 80 and older, the incidence rises to 28%, while in women, it reaches 26% (66). Traditional diagnostic criteria for DISH include: (i) the presence of vertebral flowing ossifications present at a minimum of four contiguous vertebrae, (ii) preservation of disc height and a lack of significant degenerative changes at the involved vertebrae, and (iii) absence of ankylosis at the facet-joint interface, as well as the absence of sacroiliac joint erosion, sclerosis, or fusion (67).

Because of the similarity in symptoms, DISH is often misdiagnosed as ankylosing spondylitis (AS) or other degenerative diseases of the cervical, thoracic, and lumbar spines (68, 69). However, DISH patients have a much lower positivity rate for human leukocyte antigen B27 (HLA-B27) than AS patients. Additionally, DISH patients do not typically experience significant morning stiffness, which can aid in differentiating between the two conditions (70).

Nonsurgical management, including analgesics, nonsteroidal anti-inflammatory medications, and muscle relaxants, is commonly used for mild-to-moderate cases of DISH to reduce pain and preserve range of motion (71). Surgical intervention may be required for 1–15% of DISH patients experiencing worsening dysphagia or airway obstruction caused by cervical osteophyte growth (72, 73).

DISH has been recognized as a metabolic bone disorder linked to endocrine dysfunction, hyperglycemia, hyperuricemia, and obesity (67). Recent investigations have identified a genetic predisposition in the pathology of DISH, specifically through single-nucleotide polymorphisms in genes such as *FGF2* (rs1476217/rs3747676) (74), *PPP2R2D* (rs34473884) (75), *RSPO4* (rs146447064, rs14915407), *BMP4* (rs17563), and *LEMD3* (rs201930700) (76) associated with its development. However, there have been no reported miRNAs directly associated with DISH.

### Ankylosing spondylitis

AS is an autoimmune rheumatic disease that predominantly impacts the spine and sacroiliac joints, characterized by joint deformities resulting from ectopic ossification of axial joints. It has an estimated incidence rate ranging from 0.1% to 1.4% (77). Bone tissue regeneration in AS progresses rapidly, leading to the ossification of ligaments, tendons, and fascia (70). Symptoms of AS exhibit similarities to those of DISH. However, individuals with AS generally exhibit clear sacroiliitis and uveitis (78). AS demonstrates a significant genetic predisposition, particularly with a notable association with the HLA-B27 gene. Carriers of the HLA-B27:02/04/05 alleles are at a heightened risk of developing AS (79). Furthermore, various SNP loci within endoplasmic reticulum aminopeptidase-1/2, specifically rs2287987, rs10050860, rs30187, and rs27044, have been identified as conferring a protective influence in AS (80).

Therapeutic objectives for AS involve the alleviation of pain, preservation of joint mobility, and prevention of organ damage (81). Treatment modalities for AS encompass the use of nonsteroidal anti-inflammatory drugs, sulfasalazine, methotrexate, tumor necrosis factor-α inhibitors, and interleukin-17 antagonists (such as infliximab and secukinumab) (82). Additionally, it is recommended that individuals with AS cease smoking due to the potential risk of developing pulmonary fibrosis (83).

Studies have revealed that individuals with AS demonstrate increased expression of miR-29a in peripheral blood mononuclear cells compared with healthy controls (84, 85). Subsequent research has validated that miR-29a promotes the proliferation of human osteoblasts (hFOB1.19) by enhancing *Wnt* signaling through direct targeting and negative regulation of *DKK-1* (86). In comparison with the control group, individuals diagnosed with AS exhibited markedly elevated levels of miR-21. Furthermore, a negative correlation was observed between miR-21 and *PDCD4* mRNA expression (87). In the ligament tissues of AS patients, miR-124 (88) and miR-17-5p (89) were found to be upregulated. Subsequent* in vitro* studies have revealed that the use of miR-124 antagonists significantly inhibits the osteogenic differentiation of ligament fibroblasts in AS patients (88). MiR-17-5p has been demonstrated to enhance the osteogenic differentiation of ligament fibroblasts in patients with AS by targeting the ankylosis-related gene *ANKH*, leading to increased bone spur formation in rat spinal ligaments (89). Conversely, suppression of miR-17-5p has been found to ameliorate sacroiliitis inflammation in an AS rat model (89).

Additionally, beyond its role in osteogenic differentiation in AS pathogenesis, miRNAs are also involved in immune regulation in the context of AS (90). However, this particular feature is not the main focus of the present article. Recent research has revealed disparities in gut microbiota composition between AS patients and healthy individuals (91, 92). Treatment has been demonstrated to relieve dysbiosis of gut microbiota in AS patients, with potential benefits for alleviating AS symptoms (93). Hence, it has been suggested that there is an interaction between miRNAs and gut microbiota that contributes significantly to the abnormal immune response observed in AS (94).

### Spinal ligament HO

The frequency of HO in spinal ligaments was found to be notably higher in individuals of Asian descent. Specifically, the prevalence rates of ossification of the posterior longitudinal ligament (OPLL), ossification of the anterior longitudinal ligament, ossification of the ligamentum flavum (OLF), and ossification of the cervical ligament have been reported to be 6.3%, 37%, 12%, and 23%, respectively, among Japanese individuals (95), and 7.15%, 28.6%, 39.1%, and 31.5%, respectively, among Chinese individuals (96). The prevalence of ossification of the anterior longitudinal ligament and ossification of the cervical ligament was found to be higher in men than in women, whereas ossification of spinal ligaments in other areas was more prevalent in women than in men (97). However, OPLL rates are lower in North American and European patients, with a reported prevalence of 0.1–1.7% (98). Nevertheless, an archeological investigation revealed that the prevalence of thoracolumbar OLF in the Irish population was 79.9%, with a prevalence of severe OLF at 12.8% in the mid-19th century, suggesting that the incidence of OLF in European populations may have been greatly underestimated (99).

Previous research has established a correlation between the ossification of spinal ligaments and various factors, including genetics, endocrine function, mechanical stress, inflammation, and lifestyle habits (100, 101, 102, 103). Nevertheless, the precise etiology and pathogenesis of this condition remain to be fully elucidated. Treatment options for patients experiencing mild symptoms or those that can be alleviated with rest may include bed rest, head traction, physical therapy, and medication (such as pain relief and neuronutrition) (104). Surgical intervention is recommended for patients exhibiting severe symptoms, evident ossification, and spinal cord compression (105, 106).

#### Ossification of the posterior longitudinal ligament

From 2016 to 2022, a total of 14 studies reported the influence of miRNA on the OH occurrence of OPLL or the progress of OPLL patients (Supplementary Table 1). Some of these studies used public datasets (GSE5464 (107) and GSE69787 (108)) from the Gene Expression Omnibus database as the research object and used bioinformatics technology to identify a significant number of miRNAs associated with OPLL. Other studies analyzed ligament (109, 110) or blood (111, 112) samples from OPLL patients and control populations, and they also used NGS technology (112) and miRNA chip technology (113, 114) to identify miRNAs associated with OPLL. Studies have found that miR-10a-5p (target gene *ID3*) (115), miR-181a-5p (*PBX1* and *ACAN*) (116), miR-497-5p (*RSPO2*) (117), miR-320e (*TAK1*) (118), and miR-563 (*SMURF1*) (119) are upregulated in cases of OPLL. Animal experiments (115, 116, 117, 118) and *in vitro* cell experiments (119) have further confirmed that the aforementioned five miRNAs can promote the occurrence of OPLL by inhibiting the expression of their target genes. Conversely, miR-497, miR-195 (*ADORA2A*) (107), and miR-1 (long noncoding RNA (lncRNA) *MALAT1)* (120) were downregulated in cases of OPLL. *In vitro* experiments confirmed that these two miRNAs could inhibit the osteogenic differentiation of fibroblasts (107, 120).

This section includes two studies with large sample size. In the study conducted by Lim *et al*. (111), 207 patients diagnosed with OPLL and 200 control subjects were recruited. Peripheral blood samples were used to analyze the SNPs of four miRNA precursor sequences. The findings revealed a significant association between the miR-499 GG genotype and an increased risk of OPLL. Furthermore, the combination of miR-146a, miR-149, miR-196a2, and miR-499 genotypes, along with other alleles, may serve as a genetic risk factor for OPLL among the Korean population. Xu *et al*. (112) conducted a study that included 68 patients diagnosed with ossification of the OPLL, among whom 45 had intervertebral disc herniation, and 53 were considered healthy controls. The researchers used qPCR technology to analyze the expression levels of ten miRNAs in the serum/plasma samples. The findings revealed that the predictive values of miR-10a-5p, miR-563, and miR-210-3p for distinguishing between OPLL and non-OPLL patients were reflected in the area under the receiver operating characteristic curve (AUC-ROC), with corresponding values of 0.86, 0.824, and 0.801, respectively. The combined AUC value for the three microRNAs was calculated to be 0.945. The aforementioned circulating miRNA content values were found to be significant for the diagnosis of OPLL, reaffirming the potential use of circulating miRNA as a clinical early warning indicator for HO.

Most miRNAs directly bind to target mRNA, but some studies have indicated that lncRNA also plays a role in miRNA’s affecting OPLL. Yuan *et al*. (120) found that miR-1 inhibits *CX43* expression to prevent OPLL; however, when it is bound by lncRNA *MALAT1*, osteogenesis is enhanced. *MALAT1* knockdown weakened osteogenesis by releasing miR-1, suggesting that increasing miR-1 function can weaken OPLL induced by *MALAT1* overexpression. Liao *et al*. (110) found that lncRNA *XIST* expression was elevated in ligament fibroblasts of OPLL patients, potentially inhibiting miR-17-5p expression through sponging for miR-17-5p to activate the *ANHAK/BMP2/RUNX2* pathway and enhance osteogenic differentiation and mineralization.

Circular RNA (circRNA) is synthesized from linear RNA via a noncanonical splicing covalent ligation process known as reverse splicing. Because of their closed-loop structures, circRNAs exhibit higher conservation and stability than lncRNAs and miRNAs (120). Jiang *et al*. (113) used circRNA chip technology and bioinformatics analysis to identify miR-508-3p and circ_0007292 in ligament tissues from three patients with OPLL and three control subjects. Their findings suggest that circ_0007292 may enhance the expression of the osteogenesis gene *SATB2* by acting as a sponge for miR-508-3p, thereby contributing to increased HO in OPLL.

#### Ossification of the ligamentum flavum

In 2018, researchers identified 12 upregulated and six downregulated differential miRNAs in the ligament tissues of OLF patients and healthy individuals (121). They found that miRNA-342-3p may directly target ATF3 and promote osteogenic differentiation, suggesting a positive regulation of OLF by miRNA-342-3p (122). Furthermore, miRNAs identified in previous literature reports (Supplementary Table 1) were examined, revealing that miR-132-3p (*FOXO1*, *GDF5*, and *SOX6*) (123), miR-615-3p (*FOXO1* and *GDF5*) (124), miR-199b-5p (*JAG1*) (125), and miR-182 (*NAMPT*) (126) directly targeted mRNA to negatively regulate OLF following cell validation. Notably, two independent studies confirmed that miR-132-3p (123) and miR-615-3p (124) both target the same mRNA (*FOXO1* and *GDF5*).

## Acquired HO

The process of HO is commonly characterized as an aberrant tissue healing response (39). Studies have indicated that the prevalence of HO varies among different types of injuries, with rates of 23%, 53%, and 65% observed following traumatic brain injury, traumatic spinal cord injury, and wartime high-energy limb injury, respectively. Furthermore, the incidence of HO increases to 37%, 65%, and 98% in cases of upper limb bone fracture, hip replacements, and intervertebral disc replacements, respectively (1).

Imaging tests are the primary diagnostic modality for acquired HO, with X-ray and B-mode ultrasonography commonly used for detecting intermediate and advanced stages of the condition. Radionuclide scans, MRI, and CT can identify early-stage occurrences (2). However, there is a lack of both methods and biological markers for accurately predicting the onset and recurrence risks of acquired HO (127).

There is a broadly accepted belief among experts that the surgical techniques of heterotopic bone resection and joint laxation are efficacious interventions in the management of acquired HO, leading to a notable improvement in joint function. It is considered crucial to combine these surgical methods with radiotherapy, pharmacological treatments, and rehabilitative exercises to enhance treatment efficacy and reduce the likelihood of recurrence (128). Nevertheless, surgical intervention is contingent upon a definitive diagnosis, and in one study, a recurrence rate of 6% was observed among patients post-treatment (129). Surgery can be considered appropriate 6 months after a confirmed diagnosis of HO, as intervening surgically prematurely may increase the risk of recurrence (130).

Typically, nonsteroidal anti-inflammatory drugs (such as indomethacin, celecoxib, and meloxicam), bisphosphonates (sodium etidronate and sodium clodronate), and low-dose local radiotherapy are used to prevent HO following trauma or surgery (131). However, clinical research outcomes have indicated that these measures have inconsistent efficacy, and the optimal strategy for preventing and treating HO remains undetermined (39).

Since 2013, eight studies have identified multiple differential miRNAs in cases of acquired HO (Supplementary Table 1). They reported a significant reduction in the level of miR-630 in the serum of patients with HO (132). Additionally, there was a significant increase in the expression of miR-148 in muscles and miR-195 and miR-143 in ectopic bone tissue (133). *In vitro* studies have confirmed that miR-1 and miR-206 (134), as well as miR-146b-5p and miR-424 (135), can promote osteogenic differentiation. Animal experiments have subsequently confirmed that miR-21-5p (136) can promote the occurrence of acquired HO. However, miR-203 (target gene *RUNX2*) (137), miR-630 (target gene *SLUG*) (132), and miR-337-3p (target gene *NOX4* and *IRS1*) (138) have inhibitory effects on the occurrence of acquired HO.

Neurogenic HO has been observed to occur at a rate of 20–50% following craniocerebral and spinal cord trauma, and approximately 0.5–1.2% after cerebral stroke (139, 140). A bioinformatics analysis using Gene Expression Omnibus data identified 24 miRNAs that may be potentially linked to the upregulation of *XBP1* expression following acute ischemic stroke. Furthermore, the *XBP1/JAG1/Notch* pathway is proposed as a potential mechanism for the development of HO after cerebral ischemic stroke (141). Gueguen *et al*. (142) established a murine model of spinal cord injury and drug-induced HO. In this model, they observed changes in the levels of various miRNAs that are involved in promoting or inhibiting bone formation in muscle tissues following nerve injury. The team then conducted further investigations on the functions of four specific miRNAs in fibro/adipogenic progenitors from skeletal muscle. It was discovered that by inhibiting the expression of miR-20a-5p and miR-199a-5p, which are associated with osteogenesis, or by increasing the expression of anti-osteogenic miR-214-3p and miR-146a-5p, the osteogenic differentiation of fibro/adipogenic progenitors from skeletal muscle could be effectively reduced (142).

In a 2016 study involving 730 cases, qPCR was used to analyze serum samples from patients with early and late acquired HO, as well as normal populations (132). The results indicated a significant reduction in miR-630 levels in patients with both early and late HO after controlling for age and sex. Therefore, the study suggests that miR-630 could potentially serve as an early indicator of HO. This study further supports the potential use of circulating miRNA as a clinical biomarker for the early detection of HO.

## Summary and outlook

Since 2012, approximately 50 studies related to HO have been conducted, reporting more than 100 species of miRNAs linked to the development of HO (Supplementary Table 1). Among these (Table 1), three studies based on AS, SL, and acquired HO jointly reported that miR-21 played a positive role in HO (58, 87, 136). Two studies based on OPLL and acquired HO reported that miR-1 can play a bidirectional role in the occurrence of HO, depending on its target genes (120, 134). Similar to miR-1, miR-17-5p has also been reported to have this bidirectional effect (89, 110). Two studies reported that miR-10 expression decreased in the plasma and serum of SL patients (59) but increased in the plasma of OPLL patients (112). We speculate that these inconsistent results are caused by different diseases or sample types, which further indicates the highly complex mechanism by which miRNAs affect HO occurrence.

**Table 1:** The same miRNA reported in different studies.

miRNA/Disease	Year	Country	Institution	Number of		Sample	miRNA screening	miRNA exp	miRNA effect on HO	Target mRNA*	Related genes	FV		Study
				CON	PTS							Cell	Animal	
miR-21														
AS	2014	China	Chung Shan Medical University	122	122	Blood	RI: (143)	Up	(+)	–	PDCD4	–	–	( 87)
SL	2022	China	Harbin Medical University	–	–	SaoS2	RP: (144)	Up	(+)	PTEN, DKK2	–	SaoS2	–	( 58)
Ac- HO	2024	China	Shandong University	–	–	–	RI: (145), (146)	–	(+)	–	BMP4-smad	ASCs	Mouse	(136)
miR-1														
OPLL	2019	China	Second Military Medical University	16	52	pFT	BA	Down	(−)	lncRNA MALAT1	CX43	pLFs	–	(120)
Ac- HO	2020	USA	Uniformed Services University of the Health Sciences	5	5	Bone, HO	miR-NGS	Up	(+)	SOX9	–	pMPCs	–	(134)
miR-17-5p														
OPLL	2019	China	Second Military Medical University	30	30	pFT	RP: (147)	Down	(−)	–	Runx2, lncRNA, XIST, AHNAK, BMP2	pLFs	–	(110)
AS	2020	China	Guangxi Medical University	18	20	pFT	RI: (148)	Up	(+)	ANKH	–	pLFs	Rat	( 89)
miR-10														
SL	2019	China	Guizhou Medical University	10	10	Plasma	miR-NGS	Down	–	–	–	–	–	( 59)
OPLL	2019	China	Second Military Medical University	53	68	Serum, Plasma	miR-NGS	Up	Biomarkers	–	–	–	–	(112)
miR-199b-5p														
OLF	2017	China	Peking University	0	4	pFT	RI: (149)	–	(−)	JAG1	Notch	pLFs	–	(125)
OPLL	2020	China	Guangxi Medical University	–	–		GEO (GSE69787)	–	–	–	SP1, Wnt pathway, LEF1, WNT2	–	–	(108)
miR-148														
FOP	2012	Italy	G Gaslini Institute	–	–		BA	–	(−)	ACVR1	–	Hela, U2OS, C2C12	–	( 23)
FOP	2012	China	Beijing Ditan Hospital				BA	–	(−)	ACVR1	–	Hela	–	( 24)
Ac- HO	2023	Poland	University of Warsaw	0	10	Muscle Bone, HO	miR-NGS	Up	–	–	–	–	–	(133)

*Target mRNA validated by Dual-LUC.

ASCs, adipose stem cells; AC-HO, acquired HO; BA, bioinformatics analysis; CON, controls; FV, functional verification; exp, expression; GEO, Gene Expression Omnibus database; miR NGS, miRNA next generation sequencing; pFT, ligament tissue of the patient; pLFs, ligament fibroblasts of the patient; PTS, patients; RI, results reported in; RP, previous research results of this research team.

Some studies involving a substantial number of patients have validated the potential use of plasma circulating miRNA as a biomarker for SL (60, 61, 62, 63), AS (87), OPLL (111, 112), and traumatic HO (132). This finding aids in the prompt identification of these conditions, laying the foundation for investigating new preventative and diagnostic strategies for HO. Additionally, approximately one-fifth of the studies have demonstrated the impact of miRNA on HO development at the animal level, reinforcing the idea that exogenous miRNA may have a regulatory effect on organisms and suggesting potential avenues for the advancement of therapeutic interventions for HO. Therefore, we propose a bold hypothesis: Is it feasible to use these miRNAs, known for their ability to stimulate abnormal bone formation, for localized administration in the treatment of nonunion fractures and bone defects? This hypothesis requires further investigation for validation. Furthermore, advancements in molecular biology allow for a comprehensive exploration of the molecular aspects of heterotopic ossification, which is crucial for preventing and managing HO and for understanding bone pathologies and healing processes.

HO is a common clinical complication with limited treatment efficacy. Fully understanding its features and pathogenesis is imperative for establishing a precise HO prevention and treatment system. Additionally, a thorough understanding of the characteristics, mechanisms, and processes of abnormal osteogenesis in HO is favorable for better management of osteoporosis, osteogenesis imperfecta, fracture nonunion, and related conditions. This paper summarizes the classification, characteristics, etiology, and pathogenesis, as well as relevant studies on miRNA effects in HO occurrence, aiming to support advancements in HO prevention and treatment, as well as the management of other bone diseases.

## Supplementary Materials

Supplementary Table 1 Research related to miRNA & HO

## ICMJE Conflict of Interest Statement

The authors declare that there is no conflict of interest that could be perceived as prejudicing the impartiality of the research reported.

## Funding Statement

This work did not receive any specific grant from any funding agency in the public, commercial, or not-for-profit sector.
