# Supplementary material for: The role of miRNAs as biomarkers in heterotopic ossification
Source: EFORT Open Rev. 2024 Dec 2;9(12):1120–33. doi: 10.1530/EOR-22-0100 (PMC11619732; doi:10.1530/EOR-22-0100)
Supplement: Supplementary Table 1 Research related to miRNA & HO [file EOR-22-0100supplementary_table_1.pdf]

Supplementary Table 1 Research related to miRNA & HO

Diseases	Published year	n=controls /pateins	Sample	Screening method of miRNA	miRNA	Expression of miRNA in Patient or Model	Effects of miRNA on HO	Target mRNA validated by Dual-LUC	Related genes	Functional verification		Reference
										Cell	Animal	
FOP	2012	/	/	bio-FA	148b, 365, 26a	/	(-)	ACVR1	/	Hela, U2OS, C2C12	/	[1]
	2012	/	/	bio-FA	148a	/	(-)	ACVR1	/	Hela	/	[2]
POH	2023	0/2	HO and Skin tissue	miR-NGS	208b-5p, 556-5p, 518f-3p, 493-3p	up	/	/	/	/	/	[3]
					106b-5p	down	/	/	/	/	/	
SL	2022	2/2	mOB	miR-NGS	6406, 294-3p<br>302d-3p	up<br>down	/	/	/	/	/	[4]
	2018	/	/	RP	200c-3p	/	(-)	NOGGIN	/	SaoS2	/	[5]
	2022	/	SaoS2	RP (PMID 28943430）	21-5p	up	(+)	PTEN, DKK2	/	SaoS2	/	[6]
	2019	10/10	Plasma	miR-NGS	3131, 4516, 6501-5p<br>10b-5p, 4683	up<br>down	/	/	/	/	/	[7]
	2020	107/141	Plasma	RP(PMID 31256201）	4755-5p	down	(-)	CyclinD1	/	hOB	/	[8]
	2021				122-5p	down	(-)	CDK4	/	hOB	/	[9]
					7c-5p	down	(-)	CyclinD1, Wnt9a	/	hOB	/	[10]
					486-3p	down	(-)	CyclinD1, TGF-β1	/	hOB	/	[11]
AS	2014	30/30	PMBC	RO (PMID 20551325）	29a	up	/	/	/	/	/	[12]
	2017	32/38	PMBC	RO (PMID 26273623）	29a	up	/	/	/	/	/	[13]
	2019	10/10	Tissue	RO (PMID 23529662）	29a	up	(+)	DKK1	/	hFOB1.19	/	[14]
	2014	122/122	Blood	RO (PMID 21273303）	21	up	(+)	/	PDCD4	/	/	[15]
	2019	22/32	pFT	RO (PMID 25736362）	124	up	(+)	/	GSK-3β	pLFs	/	[16]
	2020	18/20	pFT	RO (PMID 29371928）	17-5p	up	(+)	ANKH	/	pLFs	Rat	[17]
OPLL	2016	3/3	pFT	miR-NGS	(144 ↑, 74 ↓)	/	/	/	/	/	/	[18]
	2016	200/207	WBC	RO (PMID 25483824）	146a, 149, 196a2, 499 (pre-miRNA)	(some SNP increase HO risk)		/	/	/	/	[19]
	2017	4/6	pFT	RP (PMID 26868491）	563	up	(+)	SMURF1	/	pLFs	/	[20]
	2018	4/25	pFT	miR-MA	487b-3p	down	(-)	/	Wnt pathway	/	/	[21]
	2018	9/12	pFT	RP (PMID 26868491）	10a-5p	up	(+)	ID3	/	pLFs	Mouse	[22]
	2019	30/30	pFT	RP (PMID 30005876）	17-5p	down	(-)	/	Runx2<br>lncRNA<br>XIST,	pLFs	/	[23]

Diseases	Published year	n=controls /pateins	Sample	Screening method of miRNA	miRNA	Expression of miRNA in Patient or Model	Effects of miRNA on HO	Target mRNA validated by Dual-LUC	Related genes	Functional verification		Referenc e
										Cell	Animal	
									AHNAK, BMP2			
	2019	53/68	Serum, Plasma	miR-NGS	10a-5p, 563, 210-3p	/	biomarkers	/	/	/	/	[24]
	2019	16/52	pFT	bio-FA	1	down	(-)	lncRNA MALAT1	CX43	pLFs	/	[25]
	2020	19/22	pFT	RP (PMID 29907859）	181a-5p	up	(+)	PBX1, ACAN	/	pLFs	Mouse	[26]
	2020	13/13	/	GEO (GSE5464）	497, 195	down	（-）	ADORA2A	/	pLFs	/	[27]
	2020	/	/	GEO (GSE69787）	520d-3p, 4782-3p 6766-3p, 199b-5p	/	/	/	SP1, Wnt pathway, LEF1, WNT2	/	/	[28]
	2021	3/3	pFT	miR-MA	508-3p	/	/	cirR 0007292	/	pLFs	/	[30]
	2021	/	/	RO (PMID 31934153）	497-5p	up	(+)	RSPO2	Wnt pathway	pLFs h/rBMSCs	Rat	[31]
	2022	12/16	pLFs	EVs-NGS	320e	up	(+)	TAK1	/	pLFs	Mouse	[32]
OLF	2016	unclear	pFT	RO (PMID 27422605）	132-3p	down	（-）	FOXO1, GDF5, SOX6	/	pLFs	/	[33]
	2017	unclear	pFT	RO (PMID 23529662）	615-3p	down	（-）	FOXO1, GDF5	/	hFOB1.19 hBMSCs pLFs	/	[34]
	2017	0/4	pFT	RO (PMID 27363340）	199b-5p	/	（-）	JAG1	Notch	pLFs	/	[35]
	2018	4/4	pFT	miR-NGS	(12↑, 16↓)	/	/	/	/	/	/	[36]
	2018	4/4	pFT	miR-NGS	342-3p	up	（+）	ATF3	/	hMSCs	/	[37]
	2018	/	/	RO (PMID 24173697）	182	down	（-）	NAMPT	/	pLFs	/	[38]
Acquire d HO	2013	0/58	Muscle	miR-MA	146b-5p, 424	up	（+）	/	/	pSMPCs	/	[39]
	2016	0/5	Bone, HO	miR-MA	203	down	（-）	RUNX2	/	hFOB1.19	Mouse	[40]
	2016	7/7(NGS) 584/146(q PCR)	Serum	miR-NGS	630	down	（-）	SLUG	/	HD-MVE C	Mouse	[41]
	2020	5/5	Bone, HO	miR-NGS	1, 206	up	（+）	SOX9	/	pMPCs	/	[42]
	2020	/	rTDSCs	miR-MA	337-3p	/	（-）	NOX4，IRS1	/	rTDSCs, hTDSCs	Rat	[43]
	2021	/	/	GEO (GSE144306)	(24↑)	/	（+）	/	/	/	/	[44]
	2023	0/10	Muscle Bone, HO	miR-NGS	148 195, 143	up up	/	/	/	/	/	[45]
	2023	/	Muscle	miR-MA	214-3p, 146a-5p	down	(-)	/	RUNX2	pFAPs	Mouse	[46]

Diseases	Published year	n=controls /pateins	Sample	Screening method of miRNA	miRNA	Expression of miRNA in Patient or Model	Effects of miRNA on HO	Target mRNA validated by Dual-LUC	Related genes	Functional verification		Reference
										Cell	Animal	
					20a-5p, 199a-5p	up	(+)		RUNX2, OSX			
	2024	/	/	RO (PMID 34596354, 35757032）	21-5p	/	(+)	/	BMP4-smad	ASCs	Mouse	[47]

bio FA bioinformatics analysis
miR MA miRNA microarray analysis
miR NGS miRNA next generation sequencing
GEO Gene Expression Omnibus data base
RP previous research results of this research team
RO results reported in other studies
ASCs adipose stem cells
pSMPCs skeletal muscle progenitor cells of patient
rTDSCs tendon stem cells of rat
hTDSCs tendon stem cells of human
pBMSCs bone marrow mesenchymal stem cells of pateint
EVs NGS extracellular vesicles next generation sequencing
pFT ligament tissue of the patient
pLFs ligament fibroblasts of patient
pFAPs fibro-adipogenic progenitors of patient
h/rBMSCs bone marrow mesenchymal stem cells of human or rat
hFOB1.19 human SV40 transfected osteoblasts
hBMSCs human bone marrow mesenchymal stem cells
hMSCs human mesenchymal stem cell

Reference

[1] Mura M, Cappato S, Giacopelli F, Ravazzolo R, Bocciardi R. The role of the 3'UTR region in the regulation of the ACVR1/Alk-2 gene expression. PLoS One. 2012;7(12):e50958. doi: 10.1371/journal.pone.0050958. Epub 2012 Dec 5. PMID: 23227223; PMCID: PMC3515447.

[2] Song H, Wang Q, Wen J, Liu S, Gao X, Cheng J, Zhang D. ACVR1, a therapeutic target of fibrodysplasia ossificans progressiva, is negatively regulated by miR-148a. Int J Mol Sci. 2012;13(2):2063-2077. doi: 10.3390/ijms13022063. Epub 2012 Feb 15. PMID: 22408438; PMCID: PMC3292007.

[3] Gómez-Carballa A, Currás-Tuala MJ, Pischedda S, Cebey-López M, Gómez-Rial J, Rivero-Calle I, Pardo-Seco J, Bello X, Viz-Lasheras S, Justicia-Grande A, Montoto-Louzao J, Camino-Mera A, Ferreirós-Vidal I, Fraga M, Antúnez JR, Gómez R, Martinón-Torres F, Salas A. Multi-tissue transcriptomics of a unique monozygotic discordant twin case of severe progressive osseous heteroplasia. Genes Dis. 2023 Jun 19;11(3):100981. doi: 10.1016/j.gendis.2023.05.001. PMID: 38274377; PMCID: PMC10808913.

[4] Deng Q, Yang J, Zhouyang J, Sheng W, Gao S, Zhang Y, Haopeng L, Bingxin B, Mengting W. Preliminary screening of fluorine-stained osteoblastic apoptosis-related microRNA. Anat Rec (Hoboken). 2022 Feb;305(2):359-372. doi: 10.1002/ar.24709. Epub 2021 Jul 16. PMID: 34236144.

[5] Jiang Y, Yang Y, Wang H, Darko GM, Sun D, Gao Y. Identification of miR-200c-3p as a major regulator of SaoS2 cells activation induced by fluoride. Chemosphere. 2018 May;199:694-701. doi: 10.1016/j.chemosphere.2018.01.095. Epub 2018 Feb 20. PMID: 29471239.

[6] Guo N, Yu Y, Chu Y, Lou Q, Huang W, Wu L, Fan C, Su M, Zhang M, Yin F, Guan Z, Yang Y, Gao Y. miR-21-5p and canonical Wnt signaling pathway promote osteoblast function through a feed-forward loop induced by fluoride. Toxicology. 2022 Jan 30;466:153079. doi: 10.1016/j.tox.2021.153079. Epub 2021 Dec 20. PMID: 34942272.

[7] Wang F, Li C, Qin Y, Han X, Gao J, Zhang A, Luo P, Pan X. Analysis of the microRNA Profile of Coal-Burning Endemic Fluorosis Using Deep Sequencing and Bioinformatic Approaches. Bull Environ Contam Toxicol. 2019 Jul;103(1):56-63. doi: 10.1007/s00128-019-02660-8. Epub 2019 Jun 29. PMID: 31256201.

[8] Gao J, Qin Y, Luo K, Wang X, Yu C, Zhang A, Pan X. Downregulation of miR-4755-5p promotes fluoride-induced osteoblast activation via tageting Cyclin D1. J Trace Elem Med Biol. 2020 Dec;62:126626. doi: 10.1016/j.jtemb.2020.126626. Epub 2020 Jul 22. PMID: 32731110.

[9] Li C, Qin Y, Ouyang T, Yao M, Zhang A, Luo P, Pan X. miR-122-5p Mediates Fluoride-Induced Osteoblast Activation by Targeting CDK4. Biol Trace Elem Res. 2021 Apr;199(4):1215-1227. doi: 10.1007/s12011-020-02239-z. Epub 2020 Jun 22. PMID: 32572801.

[10] Luo K, Qin Y, Ouyang T, Wang X, Zhang A, Luo P, Pan X. Let-7c-5p Regulates CyclinD1 in Fluoride-Mediated Osteoblast Proliferation and Activation. Toxicol Sci. 2021 Aug 3;182(2):275-287. doi: 10.1093/toxsci/kfab054. PMID: 33982124.

[11] Ouyang T, Qin Y, Luo K, Han X, Yu C, Zhang A, Pan X. miR-486-3p regulates CyclinD1 and promotes fluoride-induced osteoblast proliferation and activation. Environ Toxicol. 2021 Sep;36(9):1817-1828. doi: 10.1002/tox.23302. Epub 2021 Jun 3. PMID: 34080770.

[12] Huang J, Song G, Yin Z, Luo X, Ye Z. Elevated miR-29a expression is not correlated with disease activity index in PBMCs of patients with ankylosing spondylitis. Mod Rheumatol. 2014 Mar;24(2):331-4. doi: 10.3109/14397595.2013.854077. PMID: 24593209.

[13] Huang J, Song G, Yin Z, Fu Z, Ye Z. MiR-29a and Messenger RNA Expression of Bone Turnover Markers in Canonical Wnt Pathway in Patients with Ankylosing Spondylitis. Clin Lab. 2017 May 1;63(5):955-960. doi: 10.7754/Clin.Lab.2017.161214. PMID: 28627829.

[14] Zhang F, Cao K, Du G, Zhang Q, Yin Z. miR-29a promotes osteoblast proliferation by downregulating DKK-1 expression and activating Wnt/β-catenin signaling pathway. Adv Clin Exp Med. 2019 Oct;28(10):1293-1300. doi: 10.17219/acem/104533. PMID: 31538414.

[15] Huang CH, Wei JC, Chang WC, Chiou SY, Chou CH, Lin YJ, Hung PH, Wong RH. Higher expression of whole blood microRNA-21 in patients with ankylosing spondylitis associated with programmed cell death 4 mRNA expression and collagen cross-linked C-telopeptide concentration. J Rheumatol. 2014 Jun;41(6):1104-11. doi: 10.3899/jrheum.130515. Epub 2014 May 1. PMID: 24786924.

[16] Tang SL, Huang QH, Wu LG, Liu C, Cai AL. MiR-124 regulates osteoblast differentiation through GSK-3β in ankylosing spondylitis. Eur Rev Med Pharmacol Sci. 2018 Oct;22(20):6616-6624. doi: 10.26355/eurrev_201810_16136. PMID: 30402833.

[17] Qin X, Zhu B, Jiang T, Tan J, Wu Z, Yuan Z, Zheng L, Zhao J. miR-17-5p Regulates Heterotopic Ossification by Targeting ANKH in Ankylosing Spondylitis. Mol Ther Nucleic Acids. 2019 Dec 6;18:696-707. doi: 10.1016/j.omtn.2019.10.003. Epub 2019 Oct 11. PMID: 31726387; PMCID: PMC6859287.

[18] Xu C, Chen Y, Zhang H, Chen Y, Shen X, Shi C, Liu Y, Yuan W. Integrated microRNA-mRNA analyses reveal OPLL specific microRNA regulatory network using high-throughput sequencing. Sci Rep. 2016 Feb 12;6:21580. doi: 10.1038/srep21580. PMID: 26868491; PMCID: PMC4751494.

[19] Lim JJ, Shin DA, Jeon YJ, Kumar H, Sohn S, Min HS, Lee JB, Kuh SU, Kim KN, Kim JO, Kim OJ, Ropper AE, Kim NK, Han IB. Association of miR-146a, miR-149, miR-196a2, and miR-499 Polymorphisms with Ossification of the Posterior Longitudinal Ligament of the Cervical Spine. PLoS One. 2016 Jul 25;11(7):e0159756. doi: 10.1371/journal.pone.0159756. PMID: 27454313; PMCID: PMC4959720.

[20] Zhang H, Xu C, Liu Y, Yuan W. [MicroRNA-563 promotes the osteogenic differentiation of posterior longitudinal ligament cells by inhibiting SMURF1]. Zhonghua Wai Ke Za Zhi. 2017

Mar 1;55(3):203-207. Chinese. doi: 10.3760/cma.j.issn.0529-5815.2017.03.008. PMID: 28241722.

[21] Yayama T, Mori K, Okumura N, Nishizawa K, Kumagai K, Nakamura A, Imai S. Wnt signaling pathway correlates with ossification of the spinal ligament: A microRNA array and immunohistochemical study. J Orthop Sci. 2018 Jan;23(1):26-31. doi: 10.1016/j.jos.2017.09.024. Epub 2017 Oct 26. PMID: 29102319.

[22] Xu C, Zhang H, Gu W, Wu H, Chen Y, Zhou W, Sun B, Shen X, Zhang Z, Wang Y, Liu Y, Yuan W. The microRNA-10a/ID3/RUNX2 axis modulates the development of Ossification of Posterior Longitudinal Ligament. Sci Rep. 2018 Jun 15;8(1):9225. doi: 10.1038/s41598-018-27514-x. PMID: 29907859; PMCID: PMC6003989.

[23] Liao X, Tang D, Yang H, Chen Y, Chen D, Jia L, Yang L, Chen X. Long Non-coding RNA XIST May Influence Cervical Ossification of the Posterior Longitudinal Ligament Through Regulation of miR-17-5P/AHNAK/BMP2 Signaling Pathway. Calcif Tissue Int. 2019 Dec;105(6):670-680. doi: 10.1007/s00223-019-00608-y. Epub 2019 Sep 11. PMID: 31511959.

[24] Xu C, Zhang H, Zhou W, Wu H, Shen X, Chen Y, Liao M, Liu Y, Yuan W. MicroRNA-10a, -210, and -563 as circulating biomarkers for ossification of the posterior longitudinal ligament. Spine J. 2019 Apr;19(4):735-743. doi: 10.1016/j.spinee.2018.10.008. Epub 2018 Oct 20. PMID: 30352301.

[25] Yuan X, Guo Y, Chen D, Luo Y, Chen D, Miao J, Chen Y. Long non-coding RNA MALAT1 functions as miR-1 sponge to regulate Connexin 43-mediated ossification of the posterior longitudinal ligament. Bone. 2019 Oct;127:305-314. doi: 10.1016/j.bone.2019.06.019. Epub 2019 Jul 4. PMID: 31280017.

[26] Liu N, Zhang Z, Li L, Shen X, Sun B, Wang R, Zhong H, Shi Q, Wei L, Zhang Y, Wang Y, Xu C, Liu Y, Yuan W. MicroRNA-181 regulates the development of Ossification of Posterior longitudinal ligament via Epigenetic Modulation by targeting PBX1. Theranostics. 2020 Jun 12;10(17):7492-7509. doi: 10.7150/thno.44309. PMID: 32685001; PMCID: PMC7359103.

[27] Jiang A, Wang N, Jiang Y, Yan X, Chen G, Chi H, Kong P, Ren H, Xia S, Ji Y, Yan J. Methylation-mediated down-regulation of microRNA-497-195 cluster confers osteogenic differentiation in ossification of the posterior longitudinal ligament of the spine via ADORA2A. Biochem J. 2020 Jun 26;477(12):2249-2261. doi: 10.1042/BCJ20200157. PMID: 32432317.

[28] Xu G, Liu C, Liang T, Qin Z, Yu CJ, Zhang Z, Jiang J, Chen J, Zhan X. Integrated miRNA-mRNA network revealing the key molecular characteristics of ossification of the posterior longitudinal ligament. Medicine (Baltimore). 2020 May 22;99(21):e20268. doi: 10.1097/MD.0000000000020268. PMID: 32481304; PMCID: PMC7249941.

[29] Jiang A, Wang N, Yan X, Jiang Y, Song C, Chi H, Chen G, Wu F, Ji Y, Yan J. Hsa-circ-0007292 promotes the osteogenic differentiation of posterior longitudinal ligament cells via regulating SATB2 by sponging miR-508-3p. Aging (Albany NY). 2021 Aug 23;13(16):20192-20217. doi: 10.18632/aging.203381. Epub 2021 Aug 23. PMID: 34483137; PMCID: PMC8436939.

[30] Chen X, Wang S, Cui Z, Gu Y. Bone marrow mesenchymal stem cell-derived extracellular vesicles containing miR-497-5p inhibit RSPO2 and accelerate OPLL. Life Sci. 2021 Aug 15;279:119481. doi: 10.1016/j.lfs.2021.119481. Epub 2021 Apr 12. PMID: 33857573.

[31] Xu C, Zhang Z, Liu N, Li L, Zhong H, Wang R, Shi Q, Zhang Z, Wei L, Hu B, Zhang H, Shen X, Wang Y, Liu Y, Yuan W. Small extracellular vesicle-mediated miR-320e transmission promotes osteogenesis in OPLL by targeting TAK1. Nat Commun. 2022 May 5;13(1):2467. doi: 10.1038/s41467-022-29029-6. PMID: 35513391; PMCID: PMC9072352.

[32] Qu X, Chen Z, Fan D, Sun C, Zeng Y, Guo Z, Qi Q, Li W. MiR-199b-5p inhibits osteogenic differentiation in ligamentum flavum cells by targeting JAG1 and modulating the Notch signalling pathway. J Cell Mol Med. 2017 Jun;21(6):1159-1170. doi: 10.1111/jcmm.13047. Epub 2016 Dec 13. PMID: 27957826; PMCID: PMC5431140.

[33] Han Y, Hong Y, Li L, Li T, Zhang Z, Wang J, Xia H, Tang Y, Shi Z, Han X, Chen T, Liu Q, Zhang M, Zhang K, Hong W, Xue Y. A Transcriptome-Level Study Identifies Changing Expression Profiles for Ossification of the Ligamentum Flavum of the Spine. Mol Ther Nucleic Acids. 2018 Sep 7;12:872-883. doi: 10.1016/j.omtn.2018.07.018. Epub 2018 Aug 7. PMID: 30161026; PMCID: PMC6120750.

[34] Han Y, Zhang K, Hong Y, Wang J, Liu Q, Zhang Z, Xia H, Tang Y, Li T, Li L, Xue Y, Hong W. miR-342-3p promotes osteogenic differentiation via targeting ATF3. FEBS Lett. 2018 Dec;592(24):4051-4065. doi: 10.1002/1873-3468.13282. Epub 2018 Nov 15. PMID: 30381822.

[35] Qu X, Chen Z, Fan D, Sun C, Zeng Y. MiR-132-3p Regulates the Osteogenic Differentiation of Thoracic Ligamentum Flavum Cells by Inhibiting Multiple Osteogenesis-Related Genes. Int J Mol Sci. 2016 Aug 20;17(8):1370. doi: 10.3390/ijms17081370. PMID: 27556448; PMCID: PMC5000765.

[36] Yin J, Zhuang G, Zhu Y, Hu X, Zhao H, Zhang R, Guo H, Fan X, Cao Y. MiR-615-3p inhibits the osteogenic differentiation of human lumbar ligamentum flavum cells via suppression of osteogenic regulators GDF5 and FOXO1. Cell Biol Int. 2017 Jul;41(7):779-786. doi: 10.1002/cbin.10780. Epub 2017 May 11. PMID: 28460412.

[37] Zhang Q, Shen Y, Jiang Y, Zhao S, Zhou D, Xu N. Overexpression of miR-182 inhibits ossification of ligamentum flavum cells by targeting NAMPT. Exp Cell Res. 2018 Jun 15;367(2):119-131. doi: 10.1016/j.yexcr.2018.03.008. Epub 2018 Mar 27. PMID: 29601800.

[38] Oishi T, Uezumi A, Kanaji A, Yamamoto N, Yamaguchi A, Yamada H, Tsuchida K. Osteogenic differentiation capacity of human skeletal muscle-derived progenitor cells. PLoS One. 2013;8(2):e56641. doi: 10.1371/journal.pone.0056641. Epub 2013 Feb 14. PMID: 23457598; PMCID: PMC3572948.

[39] Tu B, Liu S, Yu B, Zhu J, Ruan H, Tang T, Fan C. miR-203 inhibits the traumatic heterotopic ossification by targeting Runx2. Cell Death Dis. 2016 Oct 27;7(10):e2436. doi: 10.1038/cddis.2016.325. PMID: 27787524; PMCID: PMC5133990.

[40] Sun Y, Cai J, Yu S, Chen S, Li F, Fan C. MiR-630 Inhibits Endothelial-Mesenchymal Transition by Targeting Slug in Traumatic Heterotopic Ossification. Sci Rep. 2016 Mar 4;6:22729. doi: 10.1038/srep22729. PMID: 26940839; PMCID: PMC4778133.

[41] de Vasconcellos JF, Jackson WM, Dimtchev A, Nesti LJ. A microRNA Signature for Impaired Wound-Healing and Ectopic Bone Formation in Humans. J Bone Joint Surg Am. 2020 Nov 4;102(21):1891-1899. doi: 10.2106/JBJS.19.00896. PMID: 32858559.

[42] Geng Y, Zhao X, Xu J, Zhang X, Hu G, Fu SC, Dai K, Chen X, Patrick YS, Zhang X. Overexpression of mechanical sensitive miR-337-3p alleviates ectopic ossification in rat tendinopathy model via targeting IRS1 and Nox4 of tendon-derived stem cells. J Mol Cell Biol. 2020 May 18;12(4):305-317. doi: 10.1093/jmcb/mjz030. PMID: 31065679; PMCID: PMC7232128.

[43] Chen Y, Sun Y, Xu Y, Lin WW, Luo Z, Han Z, Liu S, Qi B, Sun C, Go K, Kang XR, Chen J. Single-Cell Integration Analysis of Heterotopic Ossification and Fibrocartilage Developmental Lineage: Endoplasmic Reticulum Stress Effector Xbp1 Transcriptionally Regulates the Notch Signaling Pathway to Mediate Fibrocartilage Differentiation. Oxid Med Cell Longev. 2021 Oct 26;2021:7663366. doi: 10.1155/2021/7663366. PMID: 34737845; PMCID: PMC8563124.

[44] Mierzejewski B, Pulik Ł, Grabowska I, Sibilska A, Ciemerych MA, Łęgosz P, Brzoska E. Coding and noncoding RNA profile of human heterotopic ossifications - Risk factors and biomarkers. Bone. 2023 Nov;176:116883. doi: 10.1016/j.bone.2023.116883. Epub 2023 Aug 18. PMID: 37597797.

[45] Gueguen J, Girard D, Rival B, Fernandez J, Goriot ME, Banzet S. Spinal cord injury dysregulates fibro-adipogenic progenitors miRNAs signaling to promote neurogenic heterotopic ossifications. Commun Biol. 2023 Sep 12;6(1):932. doi: 10.1038/s42003-023-05316-w. PMID: 37700159; PMCID: PMC10497574.

[46] Wang P, Liu B, Song C, Jia J, Wang Y, Pang K, Wang Y, Chen C. Exosome MiR-21-5p Upregulated by HIF-1α Induces Adipose Stem Cell Differentiation to Promote Ectopic Bone Formation. Chem Biodivers. 2024 Apr;21(4):e202301972. doi: 10.1002/cbdv.202301972. Epub 2024 Feb 27. PMID: 38342761.
